# Detection of Potential Mutated Genes Associated with Common Immunotherapy Biomarkers in Non-Small-Cell Lung Cancer Patients

**DOI:** 10.3390/curroncol29080451

**Published:** 2022-08-15

**Authors:** Lei Cao, Zhili Cao, Hongsheng Liu, Naixin Liang, Zhongxing Bing, Caijuan Tian, Shanqing Li

**Affiliations:** 1 Department of Thoracic Surgery, Chinese Academy of Medical Sciences and Peking Union Medical College Hospital, Beijing 100730, China; 2 Tianjin Marvel Medical Laboratory, Tianjin Marvelbio Technology Co., Ltd., Tianjin 300381, China

**Keywords:** non-small-cell lung cancer, immunotherapy, tumor mutation burden, microsatellite instability, programmed cell death protein-1, gene mutation

## Abstract

Microsatellite instability (MSI), high tumor mutation burden (TMB-H) and programmed cell death 1 ligand 1 (PD-L1) expression are hot biomarkers related to the improvement of immunotherapy response. Two cohorts of non-small-cell lung cancer (NSCLC) were collected and sequenced via targeted next-generation sequencing. Drug analysis was then performed on the shared genes using three different databases: Drugbank, DEPO and DRUGSURV. A total of 27 common genes were mutated in at least two groups of TMB-H-, MSI- and PD-L1-positive groups. *AKT1*, *SMAD4*, *SCRIB* and *AXIN2* were severally involved in PI3K-activated, transforming growth factor beta (TGF-β)-activated, Hippo-repressed and Wnt-repressed pathways. This study provides an understanding of the mutated genes related to the immunotherapy biomarkers of NSCLC.

## 1. Introduction

Lung cancer is the most generally diagnosed cancer all over the world, and it is also the main cause of cancer death [1], among which NSCLC accounts for approximately 85% [2]. About 75% of patients with NSCLC are in the middle and advanced stages at the time of diagnosis, and the 5-year survival rate is less than 5%. At present, the treatment methods for advanced NSCLC patients are mainly surgery, chemotherapy and radiotherapy and other traditional tumor treatments, but the treatment effects are not satisfactory [3]. Although targeted therapies have been used in clinical treatment and have shown superior survival benefits, the ensuing drug-resistance problems are still inevitable [4]. With the development of molecular biology and immunology in recent years, immunotherapy has gradually been applied to the therapy of NSCLC, and the treatment effect is significant [5]. The characteristics and mechanism of action of immunotherapy drugs are completely different from those of traditional cytotoxic drugs and targeted drugs. Immunotherapeutic drugs do not act on tumor cells or a certain pathway, but act on the microenvironment of immunity [6]. They can effectively mobilize autoimmune function, restore cellular immune mechanisms and have an immune killing effect on the tumor. Therefore, immunotherapy is a more durable and more effective treatment, especially with the extension of treatment time, after which the treatment effect will become more obvious and patients will undergo long-term clinical remission. With the advent of immunotherapy, the 5-year survival rate of certain types of advanced NSCLC patients has increased to 16% [7], and the desire for NSCLC to become a chronic disease has gradually become a reality. As a major component of the tumor microenvironment (TME), immune infiltrates have been proven to contribute to tumor progression and immunotherapy responses [8]. Although an unprecedented durable response rate has been observed in immunotherapies, the majority of patients do not benefit from the treatment and some patients relapse after a period of response; the mechanism underlying the lack of response to immunotherapy may include adaptive immune resistance [9]. A therapeutic blockade of PD-1/PD-L1 or CTLA-4 has shown clinical benefits in only certain types of hematological malignancies such as HL, and obviously, inhibiting different immune checkpoint molecules on T cells (such as LAG-3, TIM-3 and TIGIT) can be a potential approach since these molecules inhibit T cell responses in a non-redundant manner [10]. Anti-programmed death (PD)-1 antibodies have now become the standard of care for advanced melanoma, with two drugs gaining US FDA approval in recent years: nivolumab and pembrolizumab. These have both demonstrated significant activity and a durable response with a manageable toxicity profile, and despite initial success, ongoing challenges include patient selection and predictors of response, innate resistance and the optimization of combination strategies [11].

Cancer immunotherapy, despite its long history, has come to fruition only in recent years with the advances in multiple forms of treatment, including cancer vaccines, adoptive cell transfer (ACT) and immune-checkpoint inhibitors (ICIs) [12]. In the field of tumor immunotherapy, ICIs have received the most attention [13]. The so-called immune checkpoint can be simply defined as the signaling pathway on the surface of T cells that inhibits their activation and participates in the immune response. At present, the clinical application of ICIs is mainly through competitive binding with the immunosuppressive molecule PD-L1 on the surface of tumor cells so as to block its signal transduction with the receptor PD-1 on the surface of immune cells, and reactivating T cells to play the role of immune monitoring [14]. Of course, PD-L1 inhibitors are not a panacea, and their biggest problem is that not everyone can achieve a good curative effect. PD-L1 expression status in tumor cells is an approved biomarker to predict responses to ICIs, but is not enough for optimal patient selection or to improve therapeutic outcomes. The development of novel biomarkers other than PD-L1 expression status is essential [15]. A number of factors other than PD-L1 expression, tumor infiltrating lymphocytes (TILs) and TMB, which are associated with cancer immunity, can be predictive of treatment outcomes with ICIs [12]. At present, the therapeutic effect of PD-L1 inhibitors can be predicted by biomarkers such as TMB-H [16], microsatellite instability (MSI) [17] and PD-L1 positivity [18]. Therefore, the common mutated genes associated with the three above-mentioned biomarkers are very valuable for guiding the immunotherapy of NSCLC.

In the current research, we demonstrated some potential gene-mutation biomarkers to predict the effect of immunotherapy in NSCLC patients; this gives further insights into immunotherapy and the relevant immune cell characteristics for NSCLC at the genetic level, and provides some significant indicators for predicting therapeutic effects.

## 2. Materials and Methods

### 2.1. Cohorts and Samples

#### 2.1.1. Case Inclusion Criteria

(1) Patients who were treated for the first time in our hospital and diagnosed with lung cancer; (2) persons over 18 years of age; (3) patients who did not receive radiotherapy, chemotherapy or immunotherapy before the operation; (4) patients with complete case data, having read the instructions to the subjects, and having signed a written informed consent form.

#### 2.1.2. Case Exclusion Criteria

(1) Illness combined with heart, lung or renal insufficiency, severe cerebrovascular disease or other diseases, which may affect the results; (2) patients with immune-system disorders, chronic infections or hormone use, recent antibiotic use, etc; (3) patients with an allergic constitution or allergic history to multiple drugs and patients with other cancers; (4) patients who were pregnant or lactating.

The entire study included 2 NSCLC cohorts: research group 1 and research group 2. Research group 1 covered 80 NSCLC patients who underwent targeted next-generation sequencing (NGS) of the 1000-gene panel. Research group 2 included 13 patients with NSCLC who experienced PD-L1 detection and 1000-gene-panel-targeted NGS. Adenocarcinoma of the lung was present in 44.4%, lung squamous cell carcinoma in 3.2%, adenosquamous carcinoma of the lung in 1.0% and metastasis in 13.3% of 93 patients. The tumor tissues were sequenced using targeted NGS of a 1000-cancer-gene panel, and paracancerous tissues or leukocytes were used as controls.

### 2.2. Somatic Mutation Detection 

The genome analysis toolkits GATK and VarScant were used for Indel detection [19]. Single-nucleotide variants (SNVs) were detected using MuTect and VarScan [20]. The copy number variations (CNV) were detected using CONTRA [21]. We used the self-developed fusion program to detect fusion. COSMIC (Catalogue of Somatic Mutations in Cancer, http://cancer.sanger.ac.uk/cosmic/ accessed on 12 July 2022) and OMIM (Online Mendelian Inheritance in Man, http://omin.org) were used for functional annotation. In research group 1, the TMB for each sample was calculated using the following formula: the somatic cell mutation number/the probe size (1.1 Mb). According to the TMB value, the patients were divided into TMB-H groups (>25/Mb) and low-tumor-mutation-burden (TMB-L) groups (<25/Mb). In addition, the microsatellite instability (MSI) status was also calculated, and the patients were divided into MSI (including MSI-H and MSI-L) groups and microsatellite stability (MSS) groups. According to the expression of PD-L1, the patients in group 2 were divided into PD-L1-positive (positive rate >50%) and PD-L1-negative groups (positive rate <50%).

### 2.3. Screening Specific Genes

To understand the mutated genes in different groups, according to the mutations, the unique genes of the TMB-H group, the group and the PD-L1-positive group were screened out, respectively. A Venn diagram was drawn using the VennDiagram package (version 1.6.20) in R software to show the common genes in the specific genes of the above three groups. The mutation frequencies of the common genes in patients were demonstrated using a heatmap plotted using the pheatmap package (version 1.0.12) package and ggplot2 (version 3.2.1).

### 2.4. Functional Annotation

To describe the different functions of specific genes, Gene Ontology (GO) terms and Kyoto Encyclopedia of Genes and Genomes (KEGG) pathway enrichment analysis were performed using the Database for Annotation, Visualization and Integrated Discovery (DAVID, http://david.abcc.ncifcrf.gov/ (accessed on 12 July 2022)). *p* < 0.05 was the cut-off criterion. The enrichment results were shown using ggplot2 (version 3.2.1).

### 2.5. Characteristics of Immune Microenvironment

To describe the immune microenvironment in each group, we downloaded a table of the relationships between genes and 51 immune cells and 10 immune-related pathways in published articles [22,23,24]; then, we analyzed the number of patients in each group with mutant genes in each immune cell and immune pathway. The differences in the number of people in TMB-H and TMB-L groups, MSI and MSS groups, PD-L1-positive and PD-L1-negative groups were analyzed using the Chi-square test. The pheatmap package (version 1.0.12) was adopted to produce a heatmap to show the number of people with mutations in each group. The difference analysis for each group was shown graphically using R software (version 3.4.1).

### 2.6. Drug Analysis

In order to establish a relationship between cancer drugs and genes, the Drugbank database in Metascape (http://metascape.org), the Database of Evidence for Precision Oncology (DEPO, http://dinglab.wustl.edu/depo (accessed on 12 July 2022)) and the DRUGSURV (http://www.bioprofiling.de/GEO/DRUGSURV/index.html (accessed on 12 July 2022)) online databases were used for the drug analysis of specific genes. The results for DEPO and DRUGSURV were displayed using ggplot2 (version 3.2.1) and pheatmap, respectively.

### 2.7. TCGA Verification

For the purpose of validating the above results, we further downloaded the tissue mutation data and clinical data of 5 patients with their clinical and immunotherapy information from The Cancer Genome Atlas (TCGA, https://tcga-data.nci.nih.gov/docs/publications/tcga/ (accessed on 12 July 2022) database as a validation group.

## 3. Results

### 3.1. Patients and Mutated Gene Characteristics in Different Groups

According to the TMB value, there were 43 patients in TMB-H group and 37 patients in TMB-L group, respectively. There were 105 unique mutated genes in TMB-H groups. On the other hand, group 1 also included 21 MSI and 59 MSS patients on the basis of MSI. There were 21 unique mutated genes in the MSI group. Group 2 was divided into seven patients who were PD-L1-positive and six patients who were PD-L1-negative. Compared with the negative group, the PD-L1-positive group had 80 unique genes.

**Figure 1:**
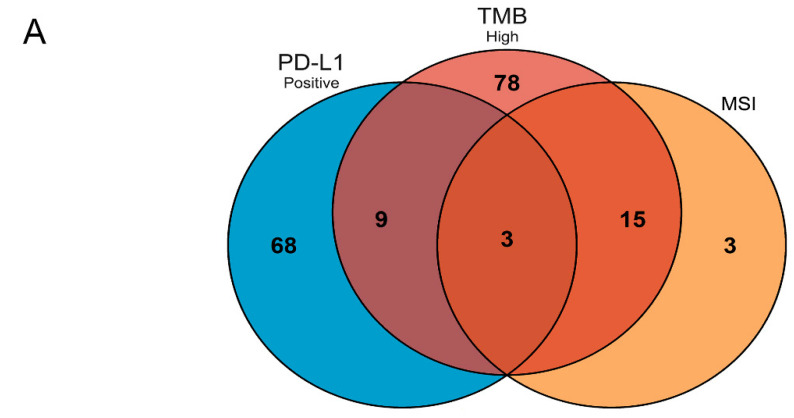

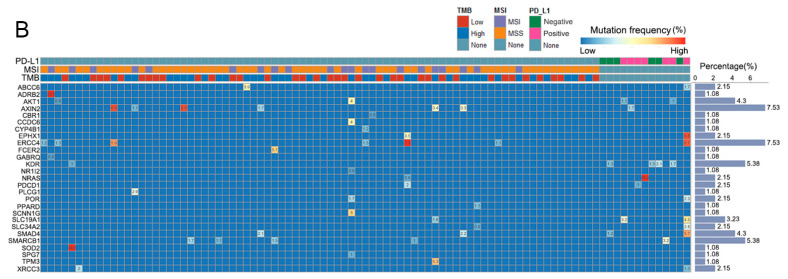
The shared mutated genes between TMB-H, MSI and PD-L1-positive groups. (**A**) Venn diagram of unique mutated gene from TMB-H, MSI and PD-L1-positive groups. There were 27 common genes in the three groups, and these genes appeared in at least two groups. (**B**) Heatmap showing the mutation frequency and percentage of 27 common genes in different groups. These genes had different mutation frequencies and percentages among these patients.

In order to understand the common mutated genes and unique mutated gene characteristics of TMB-H, MSI and PD-L1-positivity, Venn analysis was conducted on the three groups of specific genes screened above. In addition to the mutated genes unique to each group, there are 27 genes in common between the three and two groups (Figure 1A). These 27 genes were *PDCD1*, *NRAS*, *AXIN2*, *AKT1*, *SLC19A1*, *SLC34A2*, *ERCC4*, *KDR*, *SOD2*, *PLCG1*, *SMAD4*, *FCER2*, *NR1I2*, *SPG7*, *ABCC6*, *POR*, *XRCC3*, *EPHX1*, *SMARCB1*, *GABRQ*, *ADRB2*, *CCDC6*, *SCNN1G*, *CYP4B1*, *CBR1*, *TPM3* and *PPARD*. *SLC19A1*, *SLC34A2* and *POR* were the three common genes between the TMB-H, MSI and PD-L1-positive groups. Figure 1B shows the mutation frequency of these 27 genes in each sample. Both the *AXIN2* and *ERCC4* mutations accounted for the highest percentage, occurring in 7.53% of patients. All of *KDR* and *SMARCB1* were mutated in 5.38% of patients.

### 3.2. Functional Enrichment Analysis

**Figure 2:**
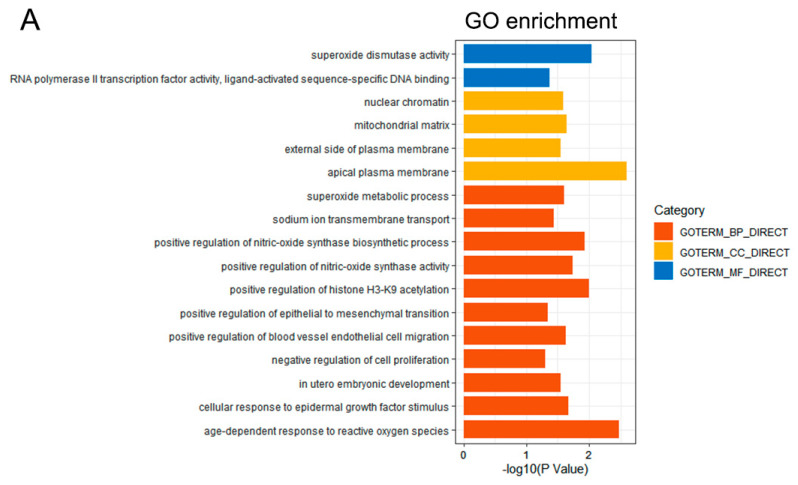

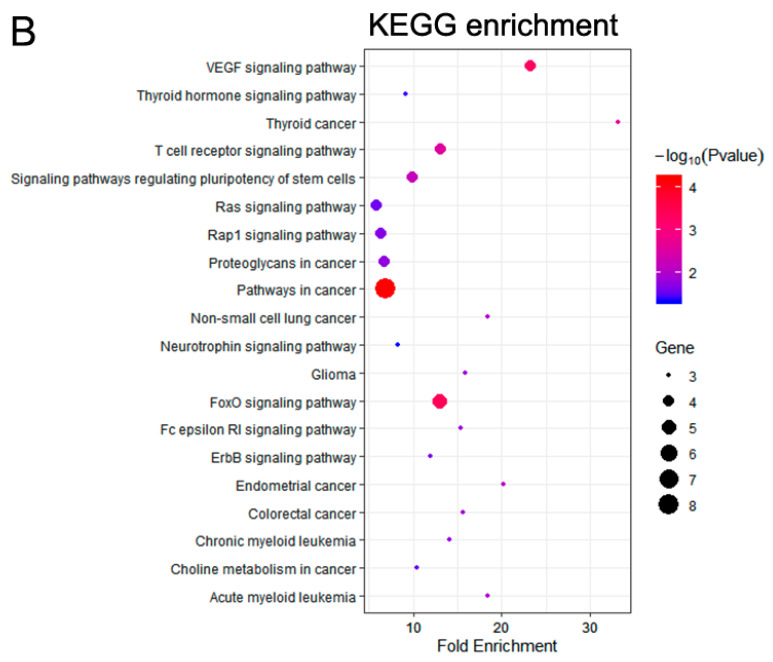
The functional enrichment analysis. (**A**) The most significant GO terms of the 27 common genes. Many studies have indicated that the great majority of GO terms are related to cancer development. (**B**) The most significant pathways of the 27 common genes. All of these pathways play a role in cancer.

GO- and KEGG-pathway enrichment analysis were conducted to analyze the 27 common genes. The 17 enriched GO terms and 20 pathways for the above genes are shown in Figure 2. From another perspective, there were 11, 4 and 2 GO terms which belonged to the biological process (BP), cellular component (CC) and molecular function (MF) categories, respectively (Figure 2A). Most GO terms were identified to be related to the occurrence and development of tumors, such as the negative regulation of cell proliferation, superoxide dismutase activity, the positive regulation of epithelial-to-mesenchymal transition, etc. All these pathways were closely associated with cancer, such as the Ras signaling pathway, the Forkhead Box Protein (*FoxO*) signaling pathway, and the vascular endothelial growth factor (*VEGF)* signaling pathway (Figure 2B).

### 3.3. Analysis of Immune Microenvironment Characteristics

**Figure 3:**
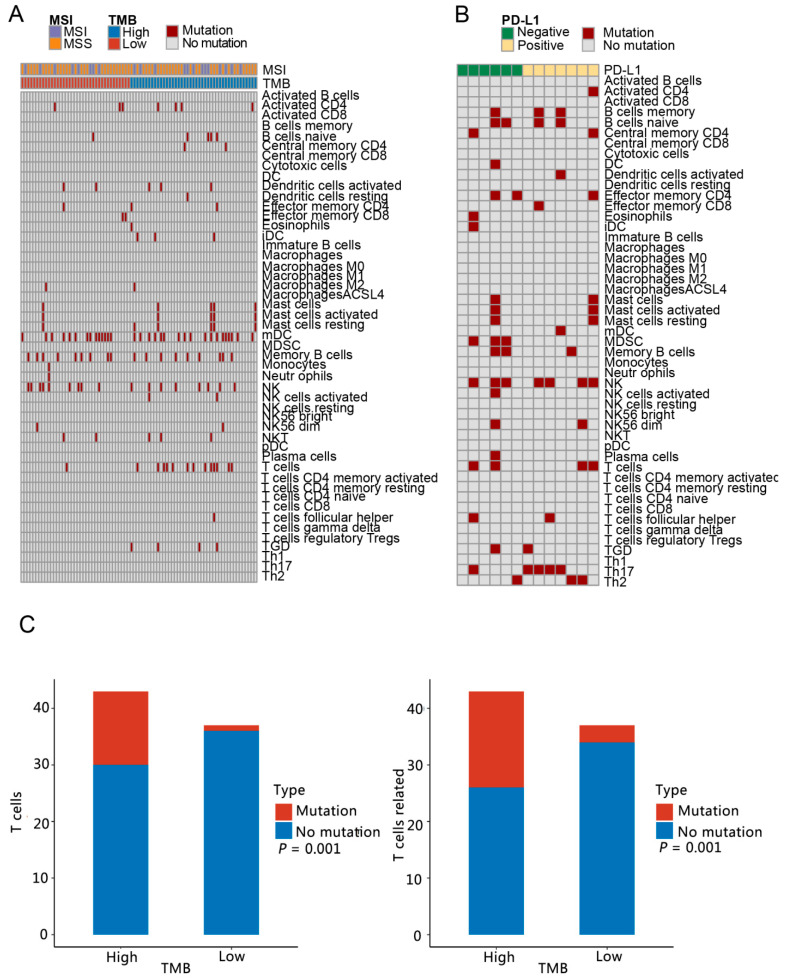
Types and functions of characteristics of immune cells in different groups. (**A**) Heatmap depicting immune cell type and function of all mutated genes in group 1. There were 25 kinds of immune cell type and function among these genes that were mutated in group 1. (**B**) Heatmap showing types and functions of immune cells of all mutated genes in group 2. These mutated genes were enriched in 26 categories of immune cell types and functions. (**C**) The number of people with the mutation genes related to immune cell type and function were compared between TMB-L and TMB-H, and there were 2 kinds of differential immune cell type and function between TMB-L and TMB-H groups.

Firstly, the types and functions of immune cells were analyzed for the number of cases with mutation genes. In group 1 and group 2, there were 25 and 26 types and functions of immune cells involved in mutation genes, respectively (Figure 3A,B). There were 20 common types and functions of immune cells in group 1 and group 2. Dendritic cells (DC) resting, macrophages M2, monocytes, neutrophils and natural killer T cells (NKT) were the immune cell types and functions unique to group 1. The specific immune cell types and functions in group 2 were memory B cells, DC, myeloid-derived suppressor cells (MDSC), plasma cells, T helper cell 17 (Th17) and T helper cell 2 (Th2). The differences in the number of people with mutation genes related to immune cells in the TMB-H and TMB-L groups, MSI and MSS groups, and PD-L1-positive and PD-L1-negative groups were analyzed, respectively. The TMB-H and TMB-L groups showed significant differences in the number of people carrying mutation genes involved in T cell and T cell-related cells (Figure 3C).

Among the 27 genes screened, according to the relationship table between genes and immune cells [13,14,15], *ADRB2* and *SMAD4* were associated with mast cells resting and central memory CD4, respectively; *FCER2* with B cells naive and DC resting; and *PDCD1* with T follicular helper cells (Tfh). However, there were no significant differences in immune cell type and function in the people with mutation genes between the TMB-H and TMB-L groups, the MSI and MSS groups and the PD-L1-positive and PD-L1-negative groups.

### 3.4. Immune Pathway Analysis

**Figure 4:**
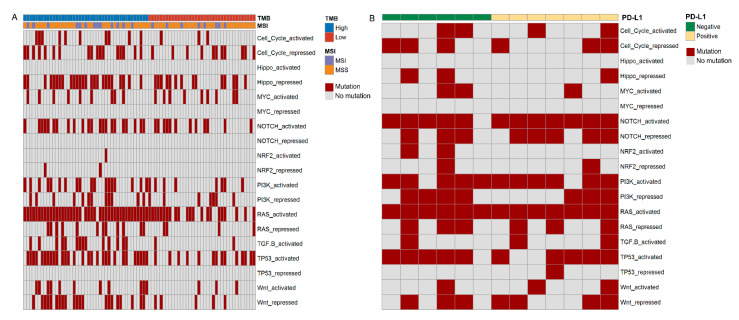
Analysis of immune-related pathway. (**A**) Heatmap depicting immune-related pathway of all mutated genes in group 1. There were 15 kinds of immune-related pathway among these genes that were mutated in group 1. (**B**) Heatmap showing immune-related pathway of all mutated genes in group 2. These mutated genes were involved in 17 categories of immune-related pathway.

**Figure 5:**
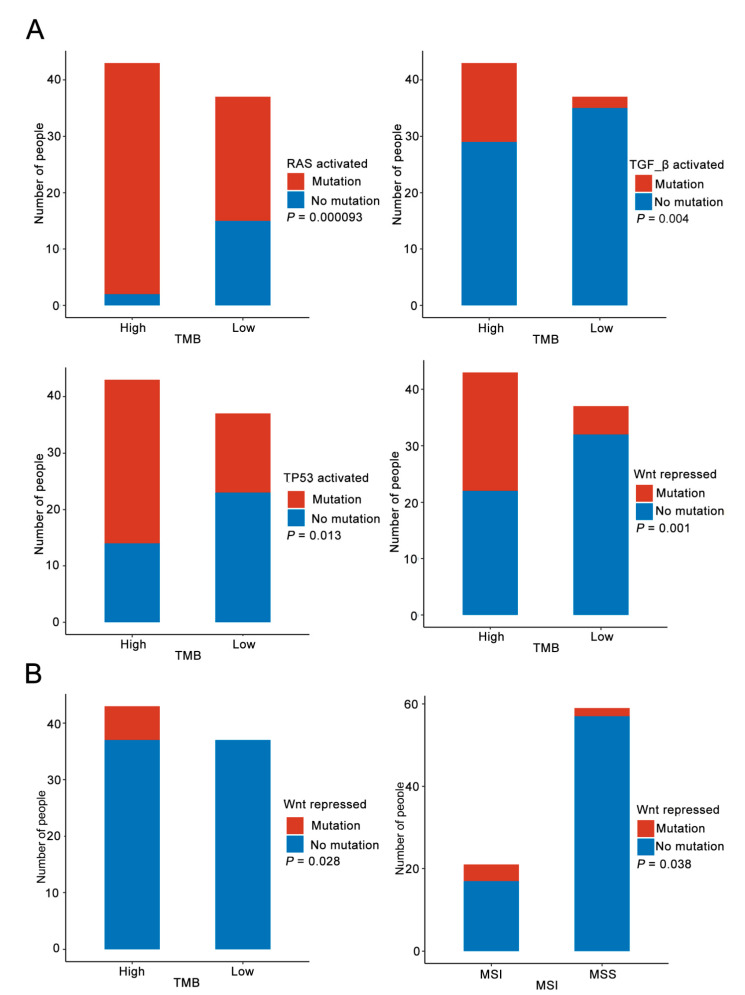
Analysis of differences in immune-related pathway. (**A**) The difference in the number of people with all the mutation genes related to RAS-activated, TGFB-activated, TP53-activated and Wnt-repressed pathway between TMB-L and TMB-H groups. (**B**) The difference in the number of people with 27 common genes related to Wnt-repressed pathway between TMB-L and TMB-H groups and MSI and MSS groups.

We analyzed the association of the mutation genes with 10 pathways related to immunity, namely cell cycle, Hippo, MYC, NOTCH, NRF2, PI3K, TGF-β, TP53, Wnt and RAS, between each group. These pathways included states of both activation and repression. In group 1 and group 2, the mutation genes happened in 15 and 17 immune-related pathways, respectively. There were 15 common pathways between these two groups, and NOTCH-repressed and TP53-repressed were the only two pathways unique to group 2 (Figure 4). RAS-activated, TGFB-activated, TP53-activated and Wnt-repressed were the four pathways that had remarkably difference in the number of people with the mutation genes between the TMB-H and TMB-L groups (Figure 5A).

In the 27 common genes, on the basis of the relationship table between the genes and immune-related pathways [22,23,24], *AKT1*, *SMAD4*, *SCRIB* and *AXIN2* were severally involved in the PI3K-activated, TGF-βactivated, Hippo-repressed and Wnt-repressed pathways. *NRAS* and *SCRIB* both participated in the RAS-activated pathway. In terms of the number of people with the related mutation genes among the 27 genes, Wnt-repressed was the only differential pathway between the TMB-H and TMB-L groups and the MSI and MSS groups (Figure 5B).

### 3.5. Drug Analysis

In order to analyze the relationship between the 27 genes screened and the tumor drugs, especially those related to immunotherapy, we conducted a drug analysis. According to the Drugbank database, *PDCD1*, *AKT1*, *POR*, *GABRQ*, *ADRB2*, *KDR*, *SOD2*, *NR1I2*, *SCNN1G*, *CBR1* and *PPARD* were targets for 140 different drugs (Table 1). The drug targets included genes such as *PDCD1,* which is targeted by nivolumab and pembrolizumab, *NR1I2* which is targeted by erlotinib, and *KDR,* which is targeted by sorafenib.

**Figure 6:**
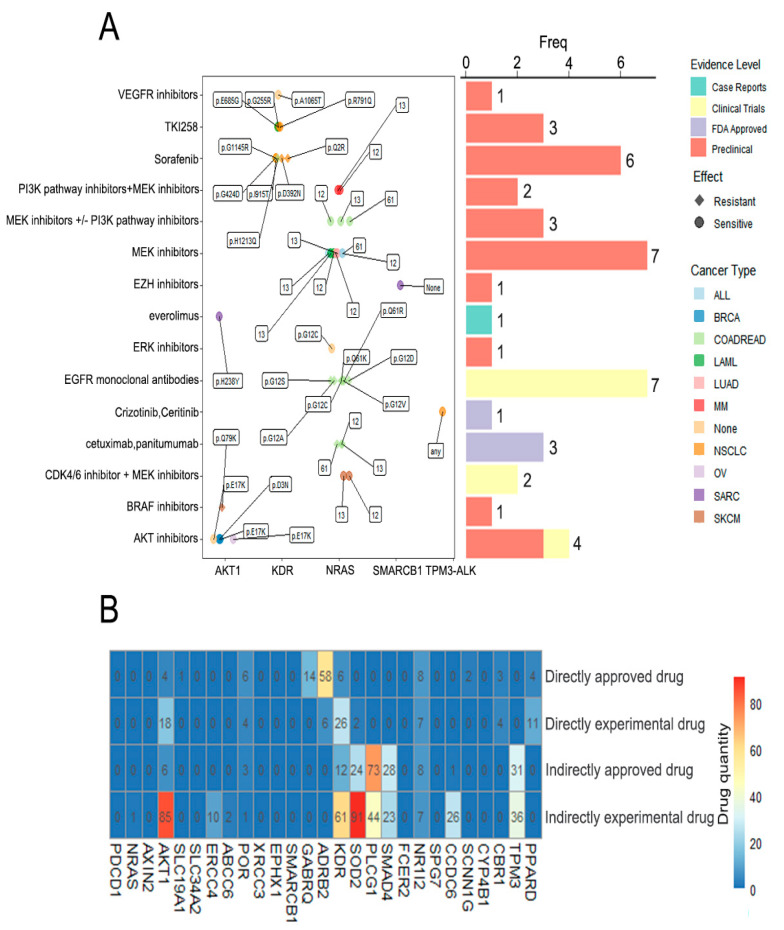
Drug analysis of 27 shared genes. (**A**) Drugs related to 27 common genes in the DEPO drug database. A total of 5 genes among these 27 common genes had related drugs. (**B**) Drugs related to 27 common genes in DRUGSURV drug database. A total of 18 genes had drugs that were directly or indirectly related.

We identified that there were 43 unique variant/drug interactions through the DEPO database. These variants were involved in five genes, namely *NRAS*, *AKT1*, *SMARCB1*, *KDR* and *TPM3-ALK.* The *NRAS* gene accounted for the largest proportion of variant/drug interactions (25/43). Six drug-associated mutations were only associated with NSCLC. Among these variant/drug interactions, four were approved by the Food and Drug Administration (FDA). MEK inhibitors and epidermal growth factor receptor (*EGFR*) monoclonal antibodies were treatments for seven different mutation sites (Figure 6A).

According to the DRUGSURV database, among these 27 genes, 9 genes had neither directly targeting drugs nor indirectly targeting drugs (Figure 6B). There were 117 kinds of drug that could be applied to *SOD2*, of which 2 were directly experimental drugs, 24 were indirectly approved drugs and 91 were indirectly experimental drugs. A total of 73 kinds of approved drug and 44 kinds of experimental drug acted indirectly on the *PLCG1*. *ADRB2*, *KDR*, *PLCG1* and *SOD2* had the most variety of directly approved drugs, directly experimental drugs, indirectly approved drugs and indirectly experimental drugs, respectively.

### 3.6. Validation of 27 Genes Screened in TCGA Database

To verify the above results, five sample patients’ clinical information and data on whether they had been treated with immunotherapy were downloaded from the TCGA database (Table 2). However, due to the small sample size, the results were not ideal. We will continue to collect relevant data for verification analysis.

## 4. Discussion

In this current study, we demonstrated the unique mutated genes of TMB-H, MSI and PD-L1-positive NSCLC patients and the common mutated genes that appeared in at least two groups; then, we further analyzed the functions, immune microenvironments and drugs associated with these genes. These data were helpful in further screening the markers related to the immunotherapy of NSCLC and to understand immune cell characteristics and their related drugs.

In the first place, 27 mutual mutated genes were recognized in TMB-H, MSI and PD-L1-positive groups. Although several studies have shown that high TMB, MSI and PD-L1-positivity can be used as predictors of response to immunotherapy, these indicators have different characteristics. Therefore, it is of great clinical significance to screen the relevant information on gene-mutation biomarkers related to these three markers. After KEGG-pathway enrichment analysis, 27 mutated genes were found to be involved in the pathways related to NSCLC, such as the VEGF signaling [25], Rap1 signaling [26], ErbB signaling [27] and Ras signaling pathways [28]. These results showed that the 27 mutated genes we filtrated were significant enough to warrant further study.

This study showed that *SLC19A1*, *SLC34A2* and *POR* were shared in the TMB-H, MSI and PD-L1-positive groups. The human solute carrier family 19, member 1 (*SLC19A1*) gene was found on chromosome 21 (21q22.3), and encoded reduced folate carrier protein 1 (RFC1), which mediates the intracellular uptake of folate. *SLC19A1* was widely expressed in human tissues and is recognized for its function in the transport of folates and anti-folate drugs [29]. The variants in the *SLC19A1* gene have been identified to be associated with many cancers, and it has been found to be related to the variable response to methotrexate (MTX) and cancer-related compounds [30]. Some research has demonstrated that pemetrexed, which was approved for its indication in NSCLC, primarily relies on *SLC19A1* to enter cells. Li et al. found that the polymorphism of the *SLC19A1* gene could predict the survival of advanced NSCLC patients treated with pemetrexed [31]. The solute carrier family 34 member 2 gene (*SLC34A2*), located on chromosome 4p15.2, is expressed in various human tissues, among which the highest expression is in lung tissue. The translation product of *SLC34A2* is NaPi-IIb, which is a type-2b sodium-dependent phosphate transporter, and plays an extremely important role in the balance of inorganic phosphorus in the body [32]. The expression of *SLC34A2* was related to cell differentiation and might play a pivotal role in tumorigenesis [33]. In NSCLC, high expression of *SLC34A2* appeared in about 3/4 of the samples and was a beneficial prognostic marker [34]. Cytochrome P450 oxidoreductase (POR) is a kind of flavin protein present on the endoplasmic reticulum membrane in human liver microsomes. It is the only donor to transfer electrons from nicotinamide adenine dinucleotide phosphate (NADPH) to all microsome Cytochrome P450 enzymes (CYP), and is responsible for the metabolism of more than 80% of clinical drugs [35]. Therefore, changes in *POR* activity caused by *POR* gene polymorphism have important clinical significance.

Then, we analyzed the microenvironment of patients in different groups. The tumor microenvironment consists of tumor cells, stromal cells (including vascular endothelial cells, pericytes, immune inflammatory cells, etc.) and the extracellular matrix. It is not only the basis for tumor growth, invasion and metastasis, but also affects the clinical treatment effect of cancers, especially immunotherapy [36]. According to the previous data [22,23,24], this study indicated that among the 27 shared genes, *ADRB2*, *SMAD4*, *FCER2* and *PDCD1* were related to different immune cell types and functions, including mast cells resting, central memory CD4, B cells naive, dendritic cells resting and Tfh [37,38,39]. Previous studies have shown that these immune cells and functions play an important role in the immune microenvironment of tumor. For instance, mast cells (MCs) are a type of granulocytic immune cells whose numbers are increased in many cancers. Previous studies have shown that the number of tumor-infiltrating mast cells is related to the increase in microvascular density, the enhancement of tumor growth and invasion and poor clinical prognosis [40]. Therefore, it is important to regulate the recruitment and activity of mast cells in malignant tumor sites for controlling the growth of tumors [41]. CD4 cells, also known as CD4-T lymphocytes, are important immune cells in the human immune system. Central memory CD4 T cells are long-term memory T cells produced by naive T cells after antigen activation. The increase in central memory CD4 T cells in peripheral blood might be used to predict the clinical response of patients with malignant melanoma to PD-1-blocking therapy [42]. Tfh cells, a specialized subgroup of CD4+T cells, play a role in inducing the activation and differentiation of B cells into immunoglobulin (Ig)-secreting cells. Ma et al. indicated that Tfh cells were likely involved in antitumor immunity and were associated with better clinical outcomes of NSCLC [43]. Therefore, in terms of regulating the tumor microenvironment, *ADRB2*, *SMAD4*, *FCER2* and *PDCD1* had vital predictive significance in the immunotherapy of NSCLC.

Similarly, the immune pathways also play an important role in immunotherapy, so we identified the relationship between the 27 common mutated genes and 10 immune pathways. Combined with previous relevant studies [22,23,24], the results of the analysis indicated that *AKT1*, *SMAD4*, *SCRIB* and *AXIN2* participated in PI3K-activated, TGF-β-activated, Hippo-repressed and Wnt-repressed pathways, respectively. Both *NRAS* and *SCRIB* took part in the RAS-activated pathway. These pathways are involved in multiple processes of the occurrence and progression of NSCLC. Among them, the PI3K pathway manages a variety of cellular functions, including transcription, translation, proliferation and apoptosis, all of which can be dysregulated in cancers such as NSCLC. The regulation of the PI3K pathway might also surmount radioresistance, chemoresistance and immune evasion in NSCLC [44]. Transforming growth factor β (TGF-β) is an important promoter of immune homeostasis and immune tolerance, and it inhibits the expansion and function of various components of the immune system [45]. The TGF-β pathway plays an important role in the occurrence and development of NSCLC [46]. It could promote the invasion and metastasis of NSCLC by promoting the epithelial–mesenchymal transition and angiogenesis, and was closely related to the drug resistance of NSCLC [47]. The Hippo pathway is a newly presented signaling pathway that plays vital roles in the development of disease [48]. In cancer cells, the dysregulation of the Hippo pathway drives numerous processes of tumor initiation and progression [49]. The Hippo pathway has been reported to be involved in the development of NSCLC and is promising as a new therapeutic target [50]. Furthermore, the Wnt and RAS pathways were also closely related to NSCLC [51,52]. Consequently, *AKT1*, *SMAD4*, *SCRIB*, *AXIN2*, *NRAS* and *SCRIB* might regulate the effects of immunotherapy by participating in immune pathways.

Because of the cost of gene sequencing, few patients chose this test. Therefore, the study has the disadvantage of an insufficient sample size. Additionally, there were 80 cases in study group 1 and 13 cases in study group 2. The sample numbers of the two groups were not uniform, resulting in the difference in the number of mutant genes in the two groups. The result of the intersection of these mutant genes needs to be further improved. Since there were only three common mutated genes in the three groups, the 27 common mutated genes appearing in the two groups were selected for subsequent analysis; thus, one group of information was lost, and the obtained results need to be further improved. In general, this study is a preliminary study, and the relevant results are exploratory; they will be further verified and improved in the follow-up research.

## 5. Conclusions

In conclusion, we have disclosed the common gene-mutation characteristics of TMB-H, MSI and PD-L1-positive NSCLC patients, which provides a unique understanding of the immunotherapy of NSCLC. This is conducive to making the diagnosis and treatment of NSCLC more personalized and accurate.

## Figures and Tables

**Table 1:** Complete list of highly correlated drug targets among the 27 common genes.

Gene Symbol	Drug(s)
*PDCD1*	CT-011; Nivolumab; Pembrolizumab
*AKT1*	Adenosine triphosphate; Arsenic trioxide; Inositol 1,3,4,5-Tetrakisphosphate; N-[2-(5-methyl-4H-1,2,4-triazol-3-yl)phenyl]-7H-pyrrolo [2,3-d]pyrimidin-4-amine; 5-(5-chloro-7H-pyrrolo[2,3-d]pyrimidin-4-yl)-4,5,6,7-tetrahydro-1H-imidazo[4,5-c]pyridine
*POR*	Flavin adenine dinucleotide; Riboflavin Monophosphate; 2′-Monophosphoadenosine 5′-Diphosphoribose
*GABRQ*	Temazepam; Midazolam; Flurazepam; Diazepam; Oxazepam; Triazolam; Ethanol; Bromazepam; Nitrazepam
*ADRB2*	Betaxolol; Isoetarine; Cabergoline; Metoprolol; Atenolol; Norepinephrine; Mirtazapine; Timolol; Phenylpropanolamine; Dipivefrin; Sotalol; Carteolol; Propranolol; Labetalol; Bisoprolol; Epinephrine; Orciprenaline; Dobutamine; Pseudoephedrine; Alprenolol; Ritodrine; Terbutaline; Bitolterol; Phenoxybenzamine; Salmeterol; Pindolol; Formoterol; Salbutamol; Isoprenaline; Arbutamine; Carvedilol; Desipramine; Acebutolol; Nadolol; Levobunolol; Metipranolol; Arformoterol; Fenoterol; Pirbuterol; Bevantolol; Penbutolol; Ephedra; Procaterol; Clenbuterol; Bambuterol; Oxprenolol; Celiprolol; Nebivolol; Indacaterol; NCX 950; Asenapine; Droxidopa; (2S)-1-(9H-Carbazol-4-yloxy)-3-(isopropylamino)propan-2-ol; Bopindolol; Bupranolol; Olodaterol; Vilanterol
*KDR*	Sorafenib; Sunitinib; N-(4-{4-AMINO-6-[4-(METHYLOXY)PHENYL]FURO[2,3-D]PYRIMIDIN-5-YL}PHENYL)-N’-[2-FLUORO-5-(TRIFLUOROMETHYL)PHENYL]UREA; AZD2171; Vatalanib; XL999; XL880; TG100801; XL820; XL184; CYC116; Ramucirumab; ABT-869; IMC-1C11; Pazopanib; Axitinib; 4-[[2-[[4-chloro-3-(trifluoromethyl)phenyl]amino]-3H-benzimidazol-5-yl]oxy]-N-methyl-pyridine-2-carboxamide; N-(4-phenoxyphenyl)-2-[(pyridin-4-ylmethyl)amino]nicotinamide; N-cyclopropyl-6-[(6,7-dimethoxyquinolin-4-yl)oxy]naphthalene-1-carboxamide; 6-chloro-N-pyrimidin-5-yl-3-{[3-(trifluoromethyl)phenyl]amino}-1,2-benzisoxazole-7-carboxamide; N-(CYCLOPROPYLMETHYL)-4-(METHYLOXY)-3-({5-[3-(3-PYRIDINYL)PHENYL]-1,3-OXAZOL-2-YL}AMINO)BENZENESULFONAMIDE; N-[5-(ETHYLSULFONYL)-2-METHOXYPHENYL]-5-[3-(2-PYRIDINYL)PHENYL]-1,3-OXAZOL-2-AMINE; 3-(2-aminoquinazolin-6-yl)-1-(3,3-dimethylindolin-6-yl)-4-methylpyridin-2(1H)-one; 3-(2-aminoquinazolin-6-yl)-4-methyl-1-[3-(trifluoromethyl)phenyl]pyridin-2(1H)-one; N’-(6-aminopyridin-3-yl)-N-(2-cyclopentylethyl)-4-methyl-benzene-1,3-dicarboxamide; N~4~-methyl-N~4~-(3-methyl-1H-indazol-6-yl)-N~2~-(3,4,5-trimethoxyphenyl)pyrimidine-2,4-diamine; N~4~-(3-methyl-1H-indazol-6-yl)-N~2~-(3,4,5-trimethoxyphenyl)pyrimidine-2,4-diamine; Cabozantinib; Regorafenib; Ponatinib; Lenvatinib; Nintedanib
*SOD2*	Benzylsulfinic Acid; 3-Fluorotyrosine
*NR1I2*	Vitamin E; Erlotinib; Estradiol; Ethinyl Estradiol; Rifampicin; Rifaximin; Paclitaxel; Docetaxel; Prasterone; Hyperforin; SR12813; N-(2,2,2-TRIFLUOROETHYL)-N-{4-[2,2,2-TRIFLUORO-1-HYDROXY-1-(TRIFLUOROMETHYL)ETHYL]PHENYL}BENZENESULFONAMIDE; Rilpivirine
*SCNN1G*	Triamterene; Amiloride
*CBR1*	2-(2-{2-[2-(2-{2-[2-(2-Ethoxy-Ethoxy)-Ethoxy]-Ethoxy}-Ethoxy)-Ethoxy]-Ethoxy}-Ethoxy)-Ethanol; Polyethyleneglycol Peg400; 3-(4-Amino-1-Tert-Butyl-1h-Pyrazolo[3,4-D]Pyrimidin-3-Yl)Phenol
*PPARD*	Icosapent; Treprostinil; Sulindac; Bezafibrate; Heptyl-Beta-D-Glucopyranoside; (11E)-OCTADEC-11-ENOIC ACID; GFT505; KD3010; (2S)-2-{3-[({[2-fluoro-4-(trifluoromethyl)phenyl]carbonyl}amino)methyl]-4-methoxybenzyl}butanoic acid; 2-({[3-(3,4-dihydroisoquinolin-2(1H)-ylsulfonyl)phenyl]carbonyl}amino)benzoic acid; 3-{5-methoxy-1-[(4-methoxyphenyl)sulfonyl]-1H-indol-3-yl}propanoic acid; {4-[3-(4-acetyl-3-hydroxy-2-propylphenoxy)propoxy]phenoxy}acetic acid

**Table 2:** The characteristics of TCGA cases.

Characteristic	TCGA-05-4402	TCGA-05-4424	TCGA-05-5425	TCGA-49-6742	TCGA-73-A9RS
Gender	Female	Male	Male	Male	Male
Age	57	70	68	70	41
Pathologic stage	Stage IV	Stage IIB	Stage IIB	Stage IIA	Stage IIB
Primary diagnosis	Adenocarcinoma with mixed subtypes	Adenocarcinoma with mixed subtypes	Adenocarcinoma with mixed subtypes	Mucinous adenocarcinoma	Adenocarcinoma, NOS
Site of resection or biopsy	Lower lobe, lung	Upper lobe, lung	Lung, NOS	Upper lobe, lung	Upper lobe, lung
Therapy drug name	Erlotinib	Erlotinib	Gefitinib Erlotinib	MDX-1106 clinical trial	Bevacizumab
Vital status	Dead	Alive	Alive	Dead	Dead

## Data Availability

The dataset used and/or analyzed during the current study is available from the corresponding author on reasonable request.
